# Does hand stiffness reflect internal organ fibrosis in diabetes mellitus?

**DOI:** 10.3389/fcdhc.2023.1198782

**Published:** 2023-07-10

**Authors:** Sanat Phatak, Jennifer L. Ingram, Pranay Goel, Satyajit Rath, Chittaranjan Yajnik

**Affiliations:** ^1^ Diabetes Unit, King Edward Memorial (KEM) Hospital Research Centre, Pune, India; ^2^ Division of Pulmonary, Allergy, and Critical Care Medicine, Department of Medicine, Duke University Medical Center, Durham, NC, United States; ^3^ Department of Biology, Indian Institute of Science Education and Research, Pune, India

**Keywords:** diabetic cheiroarthropathy, fibrosis, multi-organ, hand - pathology, joint stiffness

## Abstract

Fibrosis leads to irreversible stiffening of tissue and loss of function, and is a common pathway leading to morbidity and mortality in chronic disease. Diabetes mellitus (both type 1 and type 2 diabetes) are associated with significant fibrosis in internal organs, chiefly the kidney and heart, but also lung, liver and adipose tissue. Diabetes is also associated with the diabetic cheirarthropathies, a collection of clinical manifestations affecting the hand that include limited joint mobility (LJM), flexor tenosynovitis, Duypuytren disease and carpal tunnel syndrome. Histo-morphologically these are profibrotic conditions affecting various soft tissue components in the hand. We hypothesize that these hand manifestations reflect a systemic profibrotic state, and are potential clinical biomarkers of current or future internal organ fibrosis. Epidemiologically, there is evidence that fibrosis in one organ associates with fibrosis with another; the putative exposures that lead to fibrosis in diabetes (advanced glycation end product deposition, microvascular disease and hypoxia, persistent innate inflammation) are ‘systemic’; a common genetic susceptibility to fibrosis has also been hinted at. These data suggest that a subset of the diabetic population is susceptible to multi-organ fibrosis. The hand is an attractive biomarker to clinically detect this susceptibility, owing to its accessibility to physical examination and exposure to repeated mechanical stresses. Testing the hypothesis has a few pre-requisites: being able to measure hand fibrosis in the hand, using clinical scores or imaging based scores, which will facilitate looking for associations with internal organ fibrosis using validated methodologies for each. Longitudinal studies would be essential in delineating fibrosis trajectories in those with hand manifestations. Since therapies reversing fibrosis are few, the onus lies on identification of a susceptible subset for preventative measures. If systematically validated, clinical hand examination could provide a low-cost, universally accessible and easily reproducible screening step in selecting patients for clinical trials for fibrosis in diabetes.

## 1 Introduction

Fibrosis refers to an excessive, non-physiological accumulation of extracellular matrix (ECM) components in a body tissue (1). It leads to irreversible stiffening, a compromise in tissue function and cellular death of normal tissue. Fibrosis is a common final pathway in many disease states and is estimated to contribute to nearly half the deaths in the developed world (2). Despite considerable mechanistic understanding of the processes involved, few therapies have achieved success in reversing fibrosis (3). Until any headway is made, most impact will be made from preventive strategies, which necessitate early recognition (for effective secondary prevention) or identification of those at risk (for primary prevention).

The two major types of diabetes mellitus (henceforth, diabetes) viz type 1 (T1D) and type 2 (T2D) have been epidemiologically associated with accelerated fibrosis in various internal organs, chiefly the kidney and heart, but also the lung and liver (3–10). These conditions are common causes of morbidity and death in diabetes. Pathologic cardiac remodeling in individuals with T2D, termed, ‘diabetic cardiomyopathy,’ occurs independently of coronary heart disease and manifests as diastolic dysfunction (7). In addition, T2D is the most common cause of ‘cryptogenic’ cirrhosis in the developed world (11). A large percentage of patients with T2D have undetected liver fibrosis and cirrhosis (12). Diabetes is also an independent cause for adipose tissue fibrosis (13).

Diabetes is associated with a variety of disease manifestations occurring in the musculoskeletal system. Hand manifestations, in the form of limited joint mobility (LJM), were initially described in T1D (14, 15) but are seen in T2D as well. Diabetes is also associated with an increase in prevalence of chronic flexor tenosynovitis and trigger fingers, as well as carpal tunnel syndrome; collectively referred to as diabetic cheirarthropathy (16). Studies report marked variability in prevalence from 5 to 60%, owing partly to a heterogeneity in definition and study design (17–22). The presence of the diabetic cheirarthropathy correlates with disease duration and some micro-vascular complications but not glycated hemoglobin (HbA1C) (23, 24). However there may be stronger associations in subsets of patients: men, but not women with limited joint mobility were found to have a higher risk of retinopathy in type 1 diabetes (25). Even within diabetes related flexor tenosynovitis, retinopathy and HbA1C correlated with a particular histomorphology characterized by granulation tissue and microvascular proliferation (26).

## 2 The hypothesis

**Figure 1:**
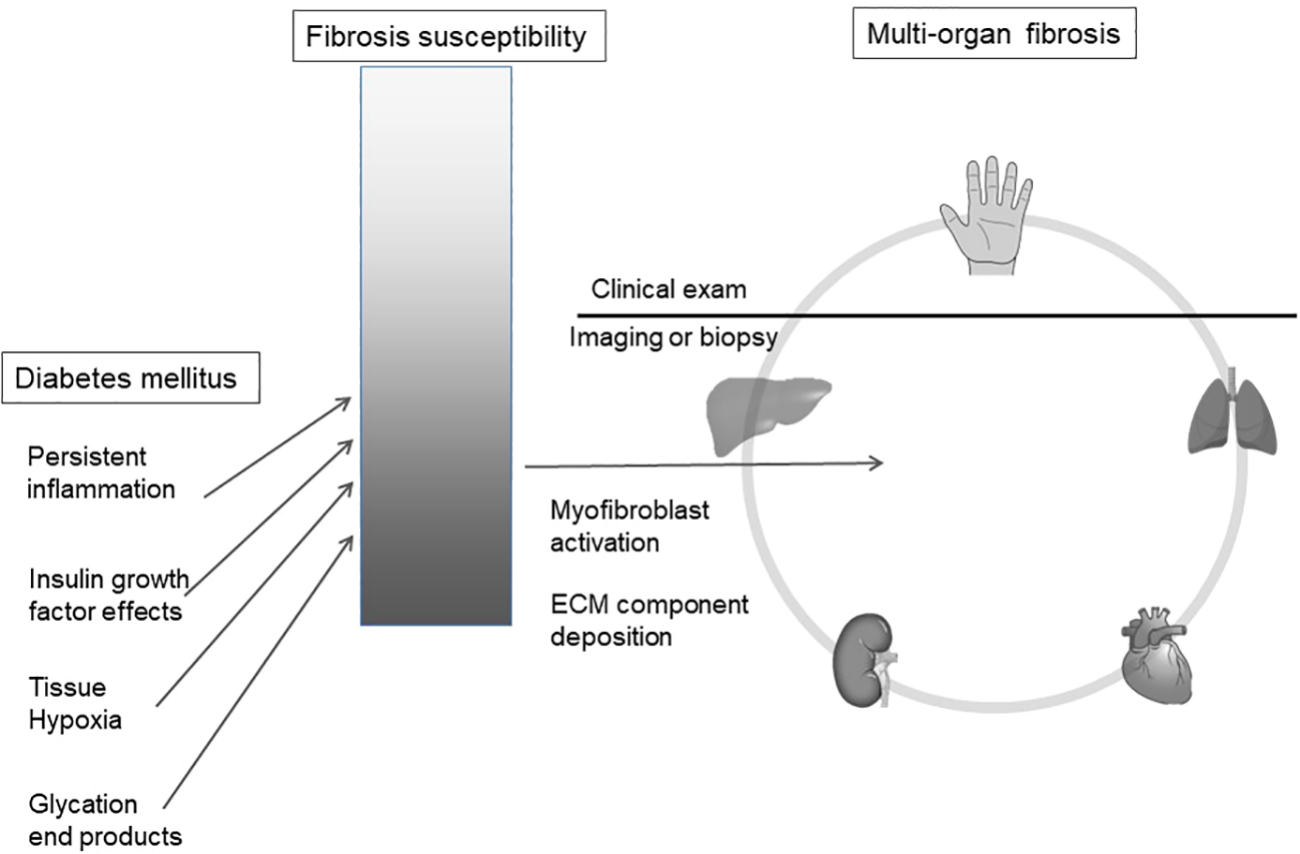
Diabetes mellitus is associated with an increased risk of organ fibrosis. We hypothesize that soft tissue fibrosis in the hand reflects internal organ fibrosis in a susceptible sub-population. ECM, extracellular matrix.

We hypothesize that observed hand manifestations in diabetes are clinical biomarkers of a susceptibility to multi-organ fibrosis. (**Figure 1**)

While diabetes is associated with profibrotic outcomes, every patient does not show these manifestations, implying that a subset of patients with diabetes are more predisposed to multi-organ fibrosis. Diabetes accelerates this underlying risk via various mechanisms such as systemic inflammation, tissue hypoxia and advanced glycation end-product deposition (27).

All the described entities occurring in the hand in diabetes are essentially fibrotic in nature; biopsy studies have demonstrated excessive collagen deposition in periarticular connective tissue including tendon sheaths and increased collagen glycation (28). Nomenclature depends on the anatomical structure in which fibrotic inflammation or thickening is observed: the palmar skin (LJM), sheaths of flexor tendons (flexor tenosynovitis) or the palmar fascia (Duypuytren’s disease, DD) (16). Regardless of nomenclature, these conditions result in preferential stiffness on the palmar aspect of the hand, thus principally limiting finger and wrist extension. In severe cases, flexion contractures ensue, leading to the ‘prayer sign’, an inability to approximate the palms fully (22).

When severe, fibrotic hand manifestations are visible on non-invasive clinical examination; even in subclinical hand involvement, hand stiffness can potentially be quantified by measuring wrist and finger extension. Since they externally represent the same process as is occurring in internal organs, we believe that hand examination may serve as a useful diagnostic or predictive biomarker for current or future multi-organ fibrotic disease.

## 3 Evaluation of the hypothesis

Although no direct evidence exists yet to demonstrate our hypothesis, we discuss several key associations that suggest a multi-organ fibrosis susceptibility in diabetes.

### 3.1 Epidemiologically, fibrosis in one organ associates with fibrosis in another organ

Individuals with a high probability of liver fibrosis using a non-invasive fibrosis score had a five-fold risk of chronic kidney disease (CKD) than those with a low probability (29) In the Multi-ethnic study of atherosclerosis (MESA), liver fibrosis - as judged by a high extracellular volume fraction (ECV) on liver T1 mapping magnetic resonance imaging (MRI)- showed an association with cardiovascular events and heart failure (30). Similarly, lung stiffness as measured on spirometry was associated with an increased risk of developing CKD (31). Such associations extend to musculoskeletal system and soft tissue fibrosis: those with Duypuytren’s disease have a three-fold risk of liver disease (20). These associations across multiple conditions and various organ systems using heterogeneous methods of assessing fibrosis, hint at an underlying, common fibrosis syndrome. The condition likely first manifests in one or two organs, with a higher likelihood in developing in others as the disease progresses.

### 3.2 Shared mechanisms suggest common pathways of fibrosis across organs

Fibrosis is histo-morphologically alike across tissues. Regardless of precipitating factors, any fibrotic process has some common pathological denominators including the presence of various extracellular proteins, laid down by essentially the same kind of cells, viz activated fibroblasts (32) Both tissue concentrations of these cells as well as sources of derivation may differ, including epithelial, mesenchymal, and endothelial (33). Fibrosis is also uniformly associated with small vessel dysfunction and hypoxia, non-infectious persistent type 2 inflammation and a consequent loss of function (34). Individual studies in multiple profibrotic situations have demonstrated a causative role of transforming growth factor – beta (TGF-B) in fibrotic processes in all organs studied (35).

### 3.3 Common genetics

Single nucleotide polymorphisms (SNPs) in TGF-B were able to predict the progression of fibrosis in chronic kidney disease (36). ‘Natural experiments’ such as short telomere syndromes suggest that genetic defects can lead to multi-organ fibrosis, commonly involving the lung and bone marrow (37). The fact that not all patients develop fibrotic manifestations in diabetes indicates a possibly quantifiable genetic risk for developing a multi-organ profibrotic state.

### 3.4 ‘Systemic’ and not local factors contribute to fibrosis in diabetes

Diabetes is associated with increase in systemic persistent inflammation (38), microvascular injury and hypoxia (39), all of which are known to stimulate profibrotic pathways. Additionally, advanced glycation end-products (AGE) stimulate ECM deposition via non-enzymatic glycosylation and subsequent collagen crosslinking, disruption of matrix-cell interactions, and interference with the renin angiotensin system (40). All of these putative mechanisms of fibrosis are ‘systemic’, implying the soft tissue of the hand would be exposed to all of these factors in the same way as the kidneys, heart and lungs, albeit with variable intensities of exposure.

Much research is ongoing about mechanisms of organ fibrosis in diabetes, and excellent reviews cover these in depth (27) Despite common exposures like hyperglycemia and insulin resistance, and common effector pathways via myofibroblast activation, multiple pathways are implicated and offer potential targets in diabetes related fibrosis. A large body of evidence suggests the central role of the TGF-B pathway and resultant fibrogenic Smad signaling (27, 41). TGF-B may also act by epithelial and endothelial -to mesenchymal transition (EndMT), both processes implicated in multiple diabetic complications (42). Hyperglycemic exposure increases the generation of cellular reactive oxygen species, and these are implicated in cell dysfunction and pathogenic profibrotic pathways (43). Profibrotic inflammatory cytokines are triggered by hyperglycemia and are likely to play a role in diabetes related fibrosis (27). Activation of the NLRP3 infalmmasome by hyperglycemia has been implicated in furthering renal and myocardial fibrosis (44). Experimentally blocking various inflammatory cytokines, such as interleukin 17, IL 6 and Tumour necrosis factor attenuate organ fibrosis and are thus likely to be involved (45–47).

Different animal models of diabetes demonstrate fibrotic responses. A rodent T1D model induced by streptozocin induces cardiac and renal fibrosis, as is also seen in leptin resistant *db/db* mice simulating T2D (48, 49). In the novel combination model of streptozocin and diet induced diabetes, fibrosis severity depends on genetic background in addition to the selection of diet (50). These heterogenous fibrosis phenotypes, despite hyperglycemia being common to all models, suggest that even in humans, hyperglycemia accelerates organ fibrosis risk in those that are already susceptible.

### 3.5 Why choose the hand as a potential clinical biomarker?

A bio-marker for a disease process is ideally rapid, inexpensive and measurable in a consistent fashion and biologically plausible. The current evidence in hand fibrosis reflecting internal organ fibrosis is scant and ambiguous. However, since it demonstrates tissue fibrosis, we believe it could be worth characterizing these associations in more detail.

A considerable amount of literature regarding the importance of mechano-sensing in initiating and propagating fibrosis now exists (51). Physical changes in the tissue micro- and macro-environment are known to induce tissue remodeling; extending the same effects likely contribute to pathological remodeling (52). Mechanosensing and consequent persistent physical stress-related signals have been implicated in the transition from ‘normal’ repair to a profibrotic process (53). Regardless of general physical activity and exercise, daily living necessitates hand use. This requirement is encapsulated by the predominance of hand-centric activities in various functional scores and indices such as the health assessment questionnaire (54). Thus, because hands are exposed to universal repetitive actions, we feel the hand is an attractive and clinically-relevant model in which to assess an underlying fibrotic state.

Tissue hypoxia is known to be a common micro-environmental factor promoting fibrosis (27). Hypoxia related fibrogenic actions commonly act via mediators such as Hypoxia inducible factor 1 (35). Distal extremities and digits are common sites that are exposed to tissue hypoxia. The fingers of the hands are common sites on which to measure hypoxemia using pulse oximetry. Finger hypoxemia correlated with increased microvascular complications such as retinopathy and nephropathy (55). The presence of diabetic hand manifestations correlates with microvascular complications (23). These associations indicate that hypoxemia, fibrosis and microvascular disease seem to be interlinked, and the hand is a potential site where all these processes are measurable.

Finally, the main advantage of looking at the hand is its clinical accessibility: soft tissues that are affected in all of these manifestations can easily be inspected and palpated non-invasively. This accessibility contrasts with the feet, where the thickness and tautness of the skin and the relatively larger subcutaneous pad of fat precludes such granular clinical examination. Hand involvement produces functional disability and typically motivates patients to seek clinical attention. In a large Taiwanese database, 9% of patients with diabetes sought medical help for one of the syndromes in the hand (18). Although these are symptomatic, it would also be useful to evaluate the predictive value of subclinical stiffness that could be systematically measured.

### 3.6 Disease models of multiple organ fibrosis: lessons from systemic sclerosis

Systemic sclerosis is an autoimmune disease characterized by inflammation, vasculopathy and multi-organ fibrosis (56). These three processes are also seen in both types of diabetes, albeit in a less severe and slower trajectory (57, 58). Although the etiologies of both these profibrotic diseases are distinct (systemic autoimmunity is not demonstrable in diabetes mellitus), putative contributors to fibrosis in both are systemic and not localized to a single organ. Organs commonly involved are the lungs, kidneys and skin and soft tissue. Hand involvement in systemic sclerosis includes skin, tenosynovial and joint inflammation and fibrosis, leading on to hand movement restrictions not unlike that seen in diabetes (59). Systemic sclerosis uses a clinical accessible site, viz the skin, as a proxy of disease severity; the two broad severity categories of the disease (limited and diffuse cutaneous systemic sclerosis) are segregated based on the extent of skin sclerosis over the upper limb. This clinical biomarker, from a simple examination, serves to prognosticate and predict fibrotic internal organ trajectories in systemic sclerosis (60). We envisage that such a principle could potentially be extrapolated to hand manifestations in diabetes mellitus.

## 4 Testing the hypothesis- where are we?

Previous studies have hinted at associations of individual fibrotic hand manifestations with diabetes complications; most have concentrated on micro- and macrovascular organ involvement (17). A systematic evaluation of hand stiffness, including subclinical asymptomatic stiffness and its association with internal organ stiffness, is, to our knowledge, yet to be done. A clinical association of hand fibrosis with various internal organ fibroses would be the first step. Within diabetes, are there patterns in organ fibrosis? Cluster analyses could potentially help classify patients with or without multi-organ fibrosis, and probe further into patterns of organ involvement within the latter group (61). Large datasets with at least one datapoint reliably conveying fibrosis in each organ of interest would be essential.

### 4.1 Longitudinal studies

‘Snapshot’ evaluations may not fully provide adequate information about multi-organ fibrosis. Despite a global susceptibility, clinically relevant fibrosis is usually seen in one organ system, often precipitated by an organ-specific injury such as infectious hepatitis in liver fibrosis (62). Well-designed longitudinal studies would be in a position to demonstrate determinants, sequence and evolution of fibrosis in organ systems. The starting point could be clinically evident fibrosis in one organ, or a cohort of those genetically susceptible to fibrosis. The hand is a potential screening tool to delineate a susceptible population in such a study.

### 4.2 Measurement of hand fibrosis

To elucidate associations in more granular detail, it would be useful to be able to quantitate the amount of fibrosis in the hand. Fibrotic manifestations suffer from vagueness of definitions and an absence of consensus; the terms, “limited joint mobility” and “prayer sign”, are at best, descriptive. Theoretically, functional hand scores such as the Cochin hand scale or the Duruoz hand index could serve as a measurable proxy for hand stiffness (63). However, these scales mostly have been used in painful hand conditions such as rheumatoid and osteo-arthritis; diabetic hand syndromes are often asymptomatic and the indices would not pick up subclinical hand stiffness restricting utility to a small fraction with severe disease.

Although none exists as yet, clinical scores in the diabetic hand measuring passive extension at various joints might encapsulate hand stiffness in a more specific and semi-quantitative manner. The HAMIS Score in systemic sclerosis does incorporate such measurements, while also including disease specific features such as digital ulcers and the modified Rodnan skin score (64).

While MRI descriptions of hand manifestations in diabetes exist (65), these are limited to case reports there has been no attempt to measure the amount of fibrotic tissue in the hand in a more systematic manner. Ultrasound could be a promising tool in this regard: an evaluation of patients with diabetic cheirarthropathy on ultrasound revealed tendon sheath thickening of more than 1 mm (66). Scores such as the Sharp/van der Heijde score or the simple erosion score in rheumatoid arthritis attempt to measure erosive and inflammatory disease activity and are now used widely in clinical trials (67). Tenosynovitis is only one manifestation under the cheirarthropathy umbrella. In order to measure hand fibrosis in diabetes, we believe imaging scores that encapsulate skin, tenosynovial and palmar fascia thickness would be important to bring more precision to a clinical quantification.

### 4.3 Multi-organ imaging

Accepted and validated non-invasive methodologies for fibrosis are different for each organ. Computed tomography (CT) scans and spirometry are used for the lung, ultrasound and liver elastography for the liver and cardiac MRI for the heart; nephrologists still depend on biopsy for renal fibrosis. These methods also assess different outcomes: elastic recoil in the liver, morphology and diastolic function in the heart, morphology in the lung. This heterogeneity makes unifying fibrosis across organ systems challenging in large studies. MRI-based modalities hold promise. The ongoing MICA and DEMISTIFI study is utilizing MRI images from the UK Biobank Registry to dissect common mechanisms in multi-organ fibrosis (68). Adding the musculoskeletal system to such an analysis would be useful in assessing its potential as a clinical biomarker of organ fibrosis. Unlike these studies that detect structural or functional results of collagen deposition, newer ‘molecular’ imaging technologies rely on processes specific to fibrosis, such as fibroblast activation or collagen deposition (69). Such imaging with probes has shown promise in systemic sclerosis: fibroblast activation on ^68^Ga-FAPI-04 PET-CT was associated with fibrosis and disease progression. These exciting new modalities could potentially be utilized to assess the fibrotic landscape in diabetes, including the hand and other soft tissue.

### 4.4 Genetic predisposition

Would examining the hand allow us to identify those who have a genetic risk of organ fibrosis? Network analysis of microarray datasets from nine different fibrotic disorders affecting different organs (such as idiopathic pulmonary fibrosis and liver cirrhosis) demonstrated common connective- tissue based networks active in all diseases, despite different manifestations (70). This finding suggests a core set of genes active in fibrosis and thus, a common genetic susceptibility to organ fibrosis. The genes that had highest upregulation such as wild-type p53-induced phosphatase 1 (*WIP1*) are involved in an inflammatory-immune response. It would be interesting to see if those with fibrotic hand manifestations demonstrate an upregulation of these common, conserved pathways.

## 5 Discussion

Fibrosis in diabetes contributes to its burden of morbidity and mortality; fibrotic diseases remain a research priority (71). Despite this emphasis on research, successes in reversing fibrosis are few. Apart from nintedanib and pirfenidone in idiopathic pulmonary fibrosis, no other antifibrotic medication is widely accepted in practice or has received regulatory approval. Cutting-edge strategies, such as the use of chimeric antigen receptor (CAR)-T cells have shown early results in targeting cardiac scar tissue (72).

Despite no established antifibrotics, many pharmacological strategies are being attempted to reverse or prevent fibrosis in diabetes, thus lending an urgency to developing a clinical outcome measure. Although tight glycemic control in itself has been reduce the progression of organ fibrosis (73), such results have not always been replicated (74). Many classes of oral agents in diabetes show evidence of antifibrotic actions in addition to that explained by their glycemic effects. Sodium Glucose Co-Transporter 2 (SGLT2) inhibitors such as empagliflozin reduce fibrosis in diabetic kidney models through AGE receptors (75); Dapagliflozin acts on NF-KB mediated inflammation and fibroblast activation, thus slowing organ fibrosis progression (76). The DPP4 inhibitor Linagliptin reduced myofibroblast conversion and reduced progression of renal fibrosis (77). Blockade of the Renin- angiotensin system also reduces fibrosis both in animal models and human participants, by various actions such as growth factor inhibition and attenuation of inflammation (78) Targeting AGEs holds promise in myocardial fibrosis and arterial stiffness (79).

Since inflammatory pathways are considered upstream of fibrosis, targeting inflammation may also have effects on diabetes related organ fibrosis. Canakinumab, a monoclonal antibody against IL-1B reduced heart failure rates, by possibly reducing myocardial remodeling (80). The anti-inflammatory endogenous peptide *N*‐acetyl‐seryl‐aspartyl‐lysyl‐proline (AcSDKP) has antifibrotic effects and is being explored for its organ protective effects (81).

We believe that advances in pharmaceutical science would be helped by establishing clinical outcome measures for trials. While genetic scores or advanced imaging technologies are potential methods to enrich cohorts by identifying those with early fibrosis and characterizing ongoing fibrosis, these approaches are developing and expensive technologies. An ideal screening tool at the population level should be inexpensive, easily reproducible. If hand fibrosis is validated rigorously as demonstrating systemic fibrosis risk, the assessment would be a valuable first step to select patients for clinical trials for up-and-coming strategies in primary and secondary prevention of fibrotic morbidity. Hand fibrosis is potentially quantifiable and could also be used as clinical outcome in such trials.

### 5.1 Future directions

The road from hypothesis to validated clinical and imaging-based outcome measures is long and winding (82). We believe that development of such a measure encapsulating the specific components of hand fibrosis would be the first step in future before being able to attempt association studies and usage in clinical trials. A detailed literature review would help find potential metrics to base the outcome measure on; the HAMIS score in systemic sclerosis is a candidate (64). We envisage that the formal development of a new instrument for diabetic hand fibrosis would require qualitative and quantitative methods, inputs from patients themselves as well as domain experts such as hand surgeons, diabetologists, neurologists and rheumatologists. It would be important to narrow the theoretical model to components that would serve as reliable indicators of fibrosis; we believe that resistance to passive stretch of select joints would be more specific for fibrosis than pain and gip strength. An involved process including testing for content validity, reliability and precision would follow before acceptance. Potential confounders such as hand osteoarthritis, coexisting inflammatory arthritis or skin disease will need due consideration. Such measures would pave the way for exploring the hypothesis using imaging and longitudinal studies.

## Data availability statement

The original contributions presented in the study are included in the article/supplementary material. Further inquiries can be directed to the corresponding authors.

## Author contributions

SP, JI, SR, PG, and CY were responsible for the hypothesis generation and manuscript planning. SP was responsible for writing. JI, SR, PG, and CY were responsible for review and editing. All authors contributed to the article and approved the submitted version.
